# Diagnosis of tuberculosis infection in children with a novel skin test and the traditional tuberculin skin test: An observational study

**DOI:** 10.1371/journal.pone.0293272

**Published:** 2024-08-27

**Authors:** Nora Fritschi, Tatyana Gureva, Platon Eliseev, Charlotte Jackson, Edith Milanzi, Siobhan Crichton, Intira Jeannie Collins, Anna Turkova, Andrei Mariandyshev, Nicole Ritz

**Affiliations:** 1 Mycobacterial and Migrant Health Research Group, University of Basel Children’s Hospital Basel and Department of Clinical Research, University of Basel, Basel, Switzerland; 2 Northern State Medical University, Arkhangelsk, Russia; 3 MRC Clinical Trials Unit at University College London, London, United Kingdom; 4 Department of Infectious Diseases, Great Ormond Street Hospital, London, United Kingdom; 5 Northern Arctic Federal University, Arkhangelsk, Russia; 6 Infectious Disease and Vaccinology Unit, University Children’s Hospital Basel, University of Basel, Basel, Switzerland; 7 Department of Pediatrics, The Royal Children’s Hospital Melbourne, The University of Melbourne, Melbourne, Australia; 8 Department of Paediatrics and Paediatric Infectious Diseases, Children’s Hospital, Lucerne Cantonal Hospital, Lucerne, Switzerland; Aurum Institute, SOUTH AFRICA

## Abstract

**Background:**

A novel skin test–called Diaskintest (DT)—containing specific *M*. *tuberculosis* antigens is in clinical use in the Russian Federation (RF). This test may improve diagnosis of tuberculosis (TB) infection. The use and performance of the DT was described and compared to the tuberculin skin test (TST).

**Methods:**

Data on children <18 years referred to a TB reference centre (Jan/2018- Dec/2019) with ≥1 DT and TST result available were analysed. An immune correlate of TB infection was defined as a positive TST (≥10 mm induration) or a positive DT (any induration).

**Results:**

Of 2710 included cases, the median age was 9.0 (IQR 5.7–13.1) years and 97.5% were BCG immunised. Overall, 1976 (79.9%) were TB uninfected, 724 (26.7%) had an immune correlate of TB infection and 10 (0.4%) TB disease. Reasons for referral were: positive or increasing skin test results in routine screening (992, 36.6%), screening before admission to a health care institution (501, 18.5%) and TB contact (457, 16.9%). DT was positive in 11.7% (308/2625) and TST in 63.1% (467/740) (Kappa 0.08, 95% CI:0.013–0.14). A positive DT was associated with older age (OR 1.16 (95% CI: 1.13–1.19) per year). Among TB contacts DT positivity was associated with contagiousness: highest proportion of positivity of 12.0% was observed when the index case was smear positive.

**Conclusion:**

In a setting with universal BCG vaccination and regular screening with TST, DT was used to rule out TB infection as TST was commonly positive. We found an association of DT positivity and contagiousness of the index case in children contacts. These observations may suggest improved specificity and sensitivity of DT compared to TST.

## Introduction

Diagnosis and treatment of tuberculosis (TB) infection and disease is emphasized by the World Health Organization (WHO) to bring TB elimination forward [1, 2]. Optimal screening approaches remain debated. Globally, the most widely used immunodiagnostic test is the tuberculin skin test (TST), which has limited specificity and positive predictive value for progression from TB infection to disease [3–5]. Skin tests using specific *Mycobacterium tuberculosis* antigens such as the Diaskintest (DT) were recently developed and are in routine clinical use in the Russian Federation (RF) [6–9].

The challenge in assessing the optimal approach for dianosing TB infection is the lack of a diagnostic gold standard [10]. TST has limited specificity in Bacillus Calmette-Guérin (BCG)-vaccinated individuals and in those exposed to non-tubercular mycobacteria [11, 12]. The characterization of the *Mycobacterium tuberculosis* genome allowed the development of more specific immunodiagnostic tests known as interferon-γ release assays (IGRA). ESAT-6/CFP-10 are the two antigens used for blood-based in-vitro stimulation in IGRAs and these have also been tested for skin tests such as the DT or the C-Tb skin test [7, 13, 14]. DT is used in children and adolescents in the RF as diagnostic and screening test but data about the performance of DT is limited, as shown by two systematic reviews and meta-analyses [15, 16]. In the more recent meta-analysis test agreement of DT with IGRA or TST was pooled from two studies of children who were assessed for TB and found a higher agreement between DT and IGRA (87.2%, 95% confidence interval (CI) 79.5–92.2) compared to the agreement of DT and TST (55.5%, 95% CI 46.1–64.5) [16].

The same meta-analysis showed in adults with microbiologically confirmed TB that the sensitivity of DT with a cut-off 5 mm induration was 91.2% (95% CI 81.7–96.0) [16]. Specificity was not reported in the meta-analysis since only studies from high TB incidence settings were available, in which TB infection was not ruled out in the control group and could thus have biased the estimate. In addition, multiple quality constraints were observed for studies evaluating DT [16]. The second meta-analysis assessed DT only and included 61 studies with over 4 million patients, of whom 98.7% were healthy individuals without TB disease [15]. The authors calculated the sensitivity of the DT in individuals with sputum smear positive TB at 86% (95% CI 80–92%) adults and 100% in children. The result should however be cautiously interpreted due to the limited sample size of sputum smear positive TB cases in the pediatric population [8, 17, 18].

Children and adolescents with TB infection are at risk of progression to TB disease [19]. Diagnosis and treatment of TB infection is emphasized to reduce the risk of TB disease and allows thus to reduce the pool of *M*. *tuberculosis* in the population. DT is likely to be associated TB infection, but none of the studies evaluating DT in children and published in the English literature so far have evaluated the performance in TB infection [8, 18].

The aim of this study was therefore to describe the use and test results of DT in children with TB infection in routine care in the Russian Federation. We aim at describing the proportions and characteristics for children diagnosed with TB infection based on DT and/or TST.

## Methods

### Study design, setting and study population

This is a retrospective observational study. Data on children and adolescents aged <18 years referred to the TB dispensary in Arkhangelsk, RF for TB screening or treatment between 1 January 2018 and 31 December 2019 were included. Excluded were children and adolescents who were referred for BCG related complications, or who had no immunodiagnostic test available.

In the RF, decentralised universal routine screening for TB infection is offered for all children annually in primary care. In addition, TB screening at a specialist TB centre, is done for children before admission to a healthcare institution (rehabilitation centres, sanatoriums), school, university or work placement. The screening approach varies with age: children 0 to 7 years are screened with a TST, children 8 to 14 years are screened with a DT and children ≥15 years are either screened with a DT for TB infection or with a chest imaging. Children with an abnormal screening result are referred to the local TB dispensary. This includes children with TST induration ≥5 mm, including a large hyperergic TST reaction, increasing TST induration compared to the previous annual test result of ≥ 6 mm, a DT with hyperaemia in the absence of induration or a DT with any size of induration [20, 21]. In addition, children requiring screening for TB infection in a targeted approach such as TB contacts or before initiation of an immunosuppressive treatment are directly referred to the TB dispensary. According to the Russian guidelines, children with an abnormal TST are re-tested with a DT.

BCG is routinely administered at birth in the RF and revaccination is considered at the age of 6 to 7 years in case of repeated negative TST results.

### Definitions

For this study TB disease was defined based on clinical diagnosis by the treating physician (based on clinical, immunological and microbiology results) and verified by documented treatment with at least three antimycobacterial drugs. For the diagnosis of TB infection in the RF variable cut-offs for skin tests are used and the increase of diameter in skin reaction in repetitive testing is an additional criterion. This is in contrast to most international definitions for TB infection, that are commonly based on a positive TST (at cut-off 5 or 10 mm) or a positive IGRA. In this analysis we therefore use the term “immune correlate of TB infection” for children with TST ≥10 mm and/or a positive DT of any size of induration. These children were further classified as treated and untreated during the study period. Children with documented negative DT, but who were given prophylactic treatment because of a contact to a TB index case in accordance with the Russian guidelines, were defined as TB uninfected. DT and TST skin reactions were defined as doubtful if redness, but no induration was observed.

### Diaskintest and tuberculin skin test

The DT and TST were done according to the national guidelines using 2 TU of PPD-L (Saint Petersburg RDE of Vaccines and Serums FMBA of Russian Federation) and 0.2 μg/0.1 ml of the Diaskintest (Generium, Russia Federation) applied intradermally and the transverse induration was measured 48 to 72 hours later [22].

### Collected data and variables

Data on all children and adolescents aged <18 years at presentation to the TB dispensary between 1 January 2018 to 31 December 2019 were collected. Individual patient level data were extracted from routine patient records, including data on demographics, diagnostics and treatment. The available immunological test results, including the tests done in primary care if available, were included for analysis.

Children who presented at the start of the study period had a longer observation period to detect progression from TB infection to TB disease. To minimize this potential observational bias, we reviewed all TB disease cases diagnosed at the TB dispensary until 31 July 2021 to identify any additional progressions. We also reviewed all records of children with immune correlate of TB infection who did not receive treatment within the studied period to determine reasons for not receiving treatment and to identify any patients who were treated between the end of the studied period (31 December 2019) and 31 July 2021.

### Statistical analysis

Analysis was restricted to children with ≥1 DT or TST available within a maximum of 91 days prior to the first visit at the dispensary. Children with doubtful results only were excluded from the analysis. For children with several test results available within the 91 days period, we selected the first DT and TST for analysis. Analyses were stratified by age in line with the age-defined screening approaches in the RF (0 to 7 years, 8 to 14 years, and ≥15 years).

For demographics median and interquartile range (IQR) were calculated as summary statistics. Comparisons between diagnosis groups were made by Chi-square for categorical and Mann-Whitney U test for continuous variables. Prevalence of positive and negative test results were calculated, and agreement was evaluated by the proportion of overall agreement (with 95% CI calculated according to Clopper and Pearson method) and Cohen’s Kappa statistic. We assessed the association between age and positive DT using logistic regression (unadjusted and adjusted for sex). Amongst patients referred as contacts of TB index cases, we also assessed the association between DT positivity and infectiousness of the index case (unconfirmed TB, smear-negative confirmed TB, smear-positive confirmed TB).

### Ethics

The study was approved by the ethics committee of the Northern State Medical University, Arkhangelsk (No3; April 20, 2020). Patients or their caregiver for all included patients gave consent for medical procedures and treatment as part or routine care and for the use of personal data for research after oral and written information. This study was retrospective, and no dedicated consent for this particular study was needed in accordance with the local ethics committee.

## Results

### Study population

**Fig 1:**
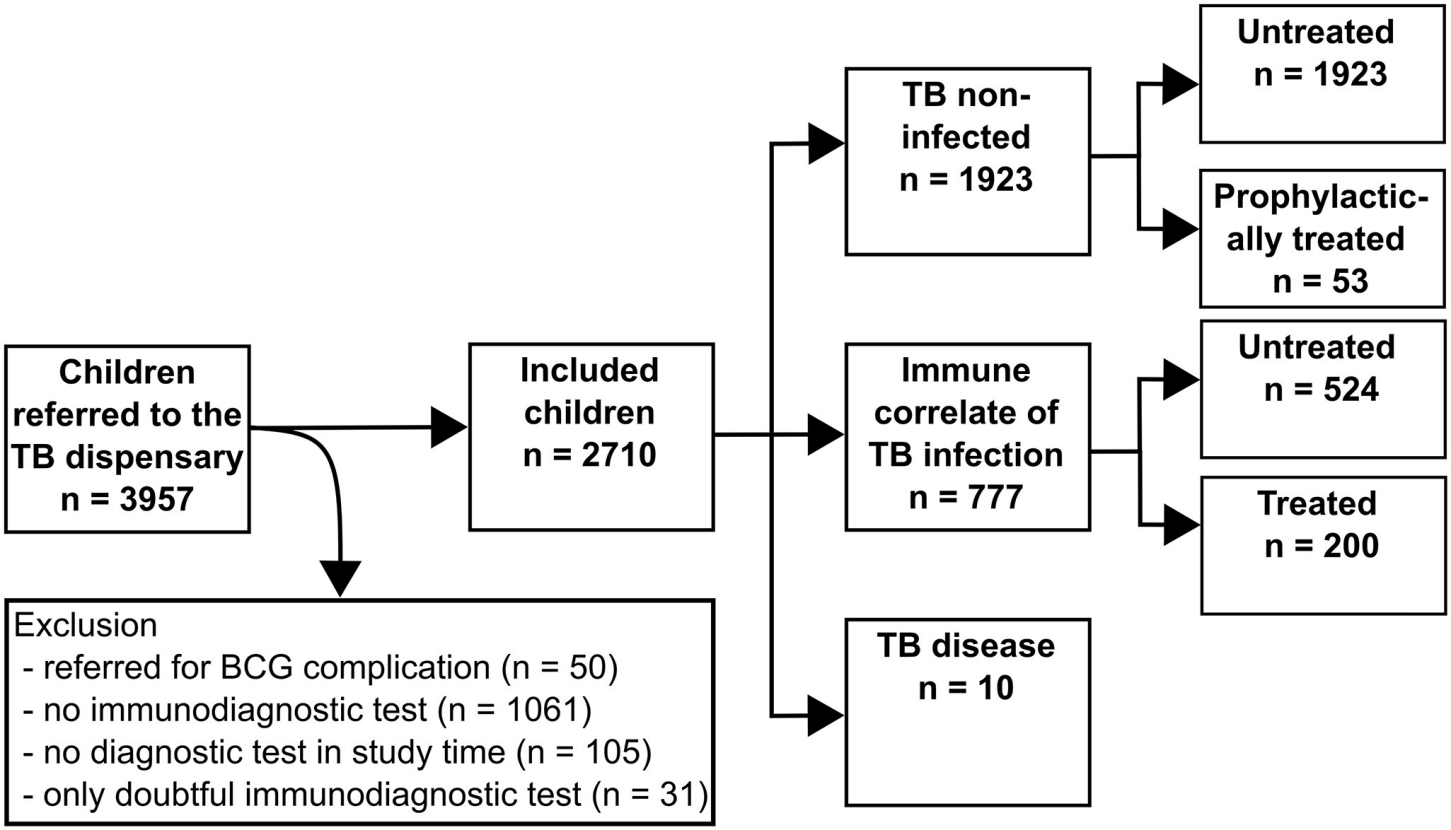
The Flow diagram shows children referred to the TB dispensary. Eligible for the final analysis were 2710 children and reasons for exclusion and numbers are displayed. Children were divided into TB disease, immune correlate of TB infection and TB non-infected. As immune correlate of TB infection, we defined for the purpose of this study the presence of at least one TST with induration of ≥10 mm and/or DT with induration of any size. Since this definition differs from the one used for TB infection and indications for TB treatment in clinical practice in the Russian Federation only a proportion of the children was started on treatment.

A total of 3957 children presented at the TB dispensary between 1 January 2018 and 31 December 2019, of whom 2710 (68.5%) met the inclusion criteria for analysis. This represents 4.1% of the total screened paediatric population in the Archangelsk region (for further data see details obtained from the Ministry of Health (S1 Fig)). Overall 1247 children were excluded from analysis and reasons are detailed in Fig 1. Children excluded from analysis compared to those included were in a similar age range, but more often male (further details in S1 Table).

Overall the median age was 9.0 (IQR 5.7–13.1) years, 1433 (52.9%) were male; 2635 (97.2%) were of European and 75 (2.8%) of Asian origin. Almost all children (98%) had received one dose of BCG vaccine. Table 1 summarises the further baseline characteristics.

**Table 1:** Characteristics of the study population in the following groups: TB-uninfected, immune correlate of TB infection with and without treatment and TB disease.

	TB uninfected, untreated (N = 1923)	TB uninfected, prophylactically treated (N = 53)	Immune correlate of TB infection, untreated (N = 524)	Immune correlate of TB infection, treated (N = 200)	TB disease (N = 10)	Total (N = 2710)
**Age (year), median (IQR)**	9.4 (5.8–13.3)	6.4 (2.8–9.8)	7.1 (5.3–10.6)	11.9 (8.7–14.3)	13.6 (11.1–15.5)	9.0 (5.7–13.1)
**Sex: male**	1038 (54.0%)	32 (60.4%)	260 (49.6%)	98 (49.0%)	5 (50.0%)	1433 (52.9%)
**BCG vaccination**						
0	23 (1.2%)	0 (0.0%)	1 (0.2%)	2 (1.0%)	0 (0.0%)	26 (1.0%)
1	1845 (97.5%)	52 (98.1%)	518 (99.4%)	192 (99.0%)	10 (100.0%)	2617 (98.0%)
2	20 (1.1%)	1 (1.9%)	2 (0.4%)	0 (0.0%)	0 (0.0%)	23 (0.9%)
at least one, but number unknown	4 (0.2%)	0 (0.0%)	0 (0.0%)	0 (0.0%)	0 (0.0%)	4 (0.1%)
Unknown	31	0	3	6	0	40
**Year**						
2018	1241 (64.5%)	23 (43.4%)	246 (46.9%)	114 (57.0%)	8 (80.0%)	1632 (60.2%)
2019	682 (35.5%)	30 (56.6%)	278 (53.1%)	86 (43.0%)	2 (20.0%)	1078 (39.8%)
**Main reason for referral**						
Abnormal annual screening	371 (19.3%)	2 (3.8%)	467 (89.1%)	151 (75.5%)	1 (10.0%)	992 (36.6%)
Admission to health institution	495 (25.7%)	0 (0.0%)	6 (1.1%)	0 (0.0%)	0 (0.0%)	501 (18.5%)
Contact with TB case	325 (16.9%)	51 (96.2%)	26 (5.0%)	48 (24.0%)	7 (70.0%)	457 (16.9%)
School entrance	140 (7.3%)	0 (0.0%)	6 (1.1%)	0 (0.0%)	0 (0.0%)	146 (5.4%)
TB-associated symptoms	125 (6.5%)	0 (0.0%)	1 (0.2%)	0 (0.0%)	1 (10.0%)	127 (4.7%)
TB investigation *	78 (4.1%)	0 (0.0%)	2 (0.4%)	0 (0.0%)	0 (0.0%)	80 (3.0%)
Changes in chest radiography	29 (1.5%)	0 (0.0%)	5 (1.0%)	0 (0.0%)	1 (10.0%)	35 (1.3%)
University/job entrance	26 (1.4%)	0 (0.0%)	3 (0.6%)	0 (0.0%)	0 (0.0%)	29 (1.1%)
Initiation of immunosuppressive treatment	19 (1.0%)	0 (0.0%)	0 (0.0%)	0 (0.0%)	0 (0.0%)	19 (0.7%)
HIV diagnosis	4 (0.2%)	0 (0.0%)	0 (0.0%)	0 (0.0%)	0 (0.0%)	4 (0.1%)
other screening reasons	311 (16.2%)	0 (0.0%)	8 (1.5%)	1 (0.5%)	0 (0.0%)	320 (11.8%)
**Follow up status at end of study time**						
Active follow up	172 (8.9%)	37 (69.8%)	49 (9.4%)	81 (40.5%)	10 (100.0%)	349 (12.9%)
Discharged	1738 (90.4%)	14 (26.4%)	465 (88.7%)	115 (57.5%)	0 (0.0%)	2332 (86.1%)
Lost to follow up	13 (0.7%)	2 (3.8%)	10 (1.9%)	4 (2.0%)	0 (0.0%)	29 (1.1%)

* TB investigation to confirm/rule out TB in children with no TB-associated symptoms.

### Reason for referral, diagnosis and treatment

Children were referred for the following reasons: 992 (36.6%) had an abnormal annual screening at primary care, 501 (18.5%) required an assessment for admission to a health care institution, 457 (16.9%) had a contact to a TB index case, 146 (5.4%) required screening for school entrance and 127 (4.7%) had symptoms suggestive of TB disease.

**Fig 2:**
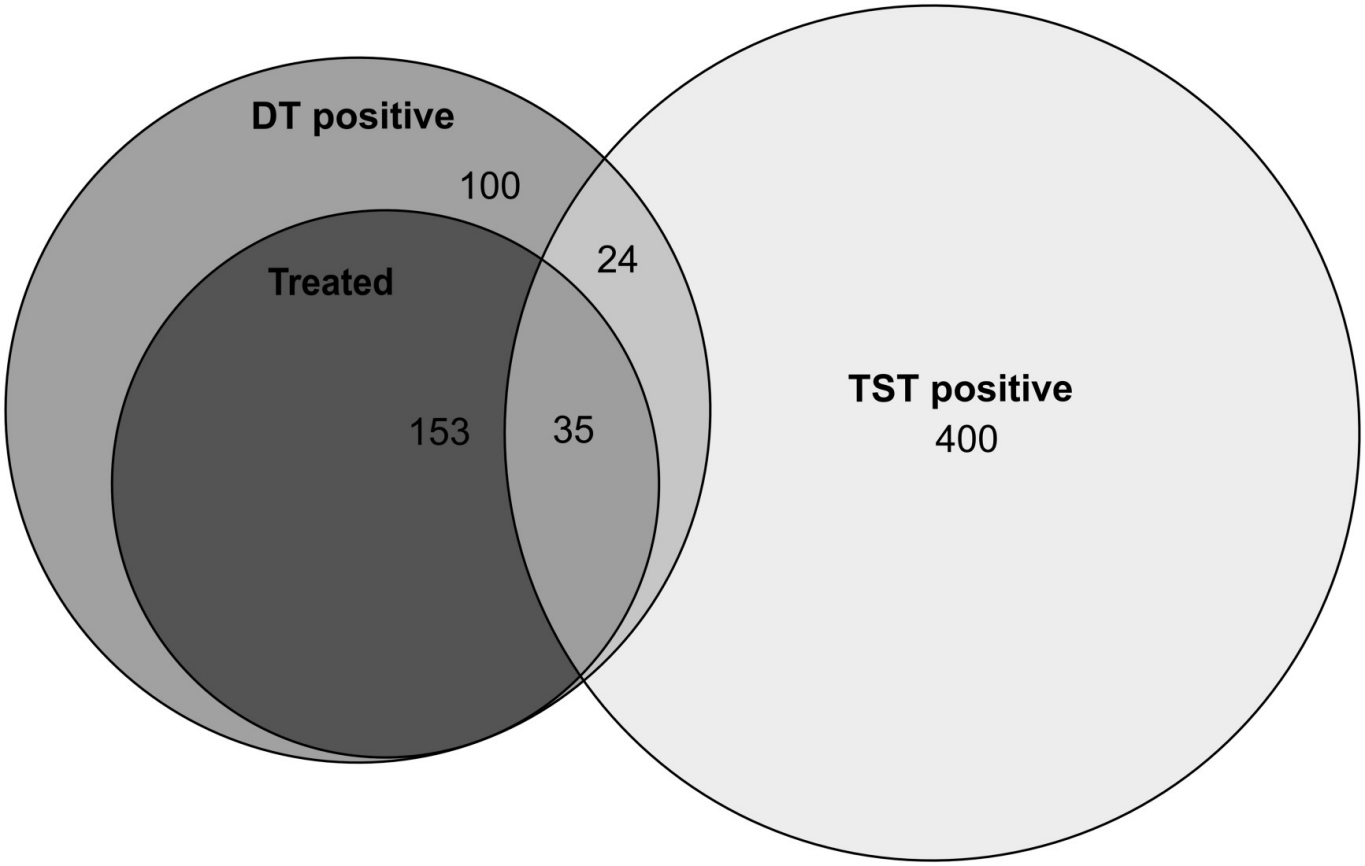
Venn diagram using exact Euler calculation showing positive test results for TST, DT and initiation of treatment in children with immune correlate of TB infection. TST positivity was defined as with induration ≥10 mm and DT positivity as any induration. The area of the ellipses is proportional to the number of cases. As immune correlate of TB infection, we defined for the purpose of this study the presence of at least one TST with induration of ≥10 mm and/or DT with induration of any size. Since this definition differs from the one used for TB infection and indications for TB treatment in clinical practice in the Russian Federation only a proportion of the children was started on treatment.

**Fig 3:**
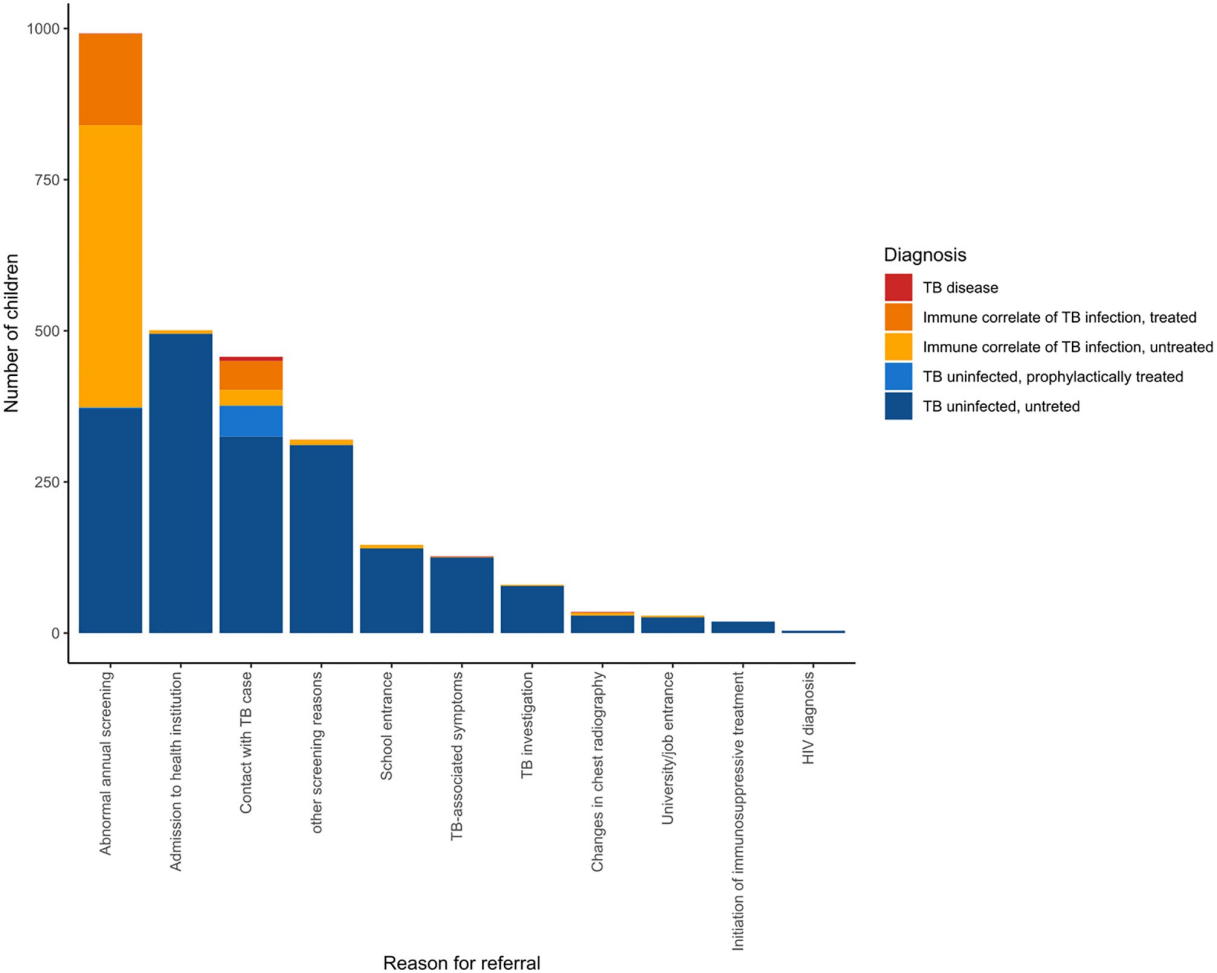
Final diagnosis of children referred to the TB dispensary by reason for referral. Among children referred for an abnormal routine annual screening and following a contact with a TB case the proportion of immune correlate of TB infection was highest with 62.3% and 16.2%, respectively (**see also** S1 Table).

Overall, 1976 (79.9%) were TB uninfected (as per the study definition), 724 (26.7%) had an immune correlate of TB infection and 10 (0.4%) had TB disease (Table 1). Of the 1976 TB uninfected children, 53 (2.8%) received prophylactic treatment and 1923 (71.0%) were untreated. Of the 724 children with immune correlate of TB infection, 200 (27.6%) were treated and 524 (72.4%) were untreated (Figs 1–3). Among the untreated children, the reasons were: a negative DT following a positive TST result in 372 (71.0%), evidence of TB infection for more than 2 years prior the current testing in 86 (16.4%), a history of treatment prior to the study period in 10 (1.9%), treatment received after the end of the studied period (31 December 2019) in 8 (1.5%), and other reasons in 48 (9.1%) children and adolescents.

The final diagnosis of all children by reason for referral is shown in S2 Table. The reason for referral by age group is shown in S2 Fig.

### DT and TST results

A total of 3696 DT and 1013 TST results were reported in the 2710 children. In total, 2018/2710 (74.5%) children had one and 601/2710 (22.2%) more than one DT reported, while 729/2710 (26.9%) children had one TST and 24/2710 (0.9%) two TST reported and 922/2710 (34.0%) had at least one of both DT and TST reported. Analysis of the first test results available per child showed: 308/2625 (11.7%) had a positive DT result and 467/740 (63.1%) a positive TST. The mean diameter of induration for DT was 1.5 (median 0, IQR 0–0) mm and 10.9 (median 11, IQR 7–15) mm for TST. Results in mm are detailed in S3 Fig. Among those tested with DT, the proportion of children with a positive result increased with age: 4.9%, 16.0% and 20.5%, in age groups 0–7 years, 8–14 years and ≥15 years, respectively. For TST no clear trend was seen: 58.8%, 78.7% and 66.7% (S4 Fig). Test results stratified by reason for referral are displayed in S5 Fig.

In a univariable logistic regression analysis a positive DT was associated with older age (OR 1.16 95% CI 1.13–1.19 per year increase). This remained similar after adjustment for sex (S3 Table).

**Fig 4:**
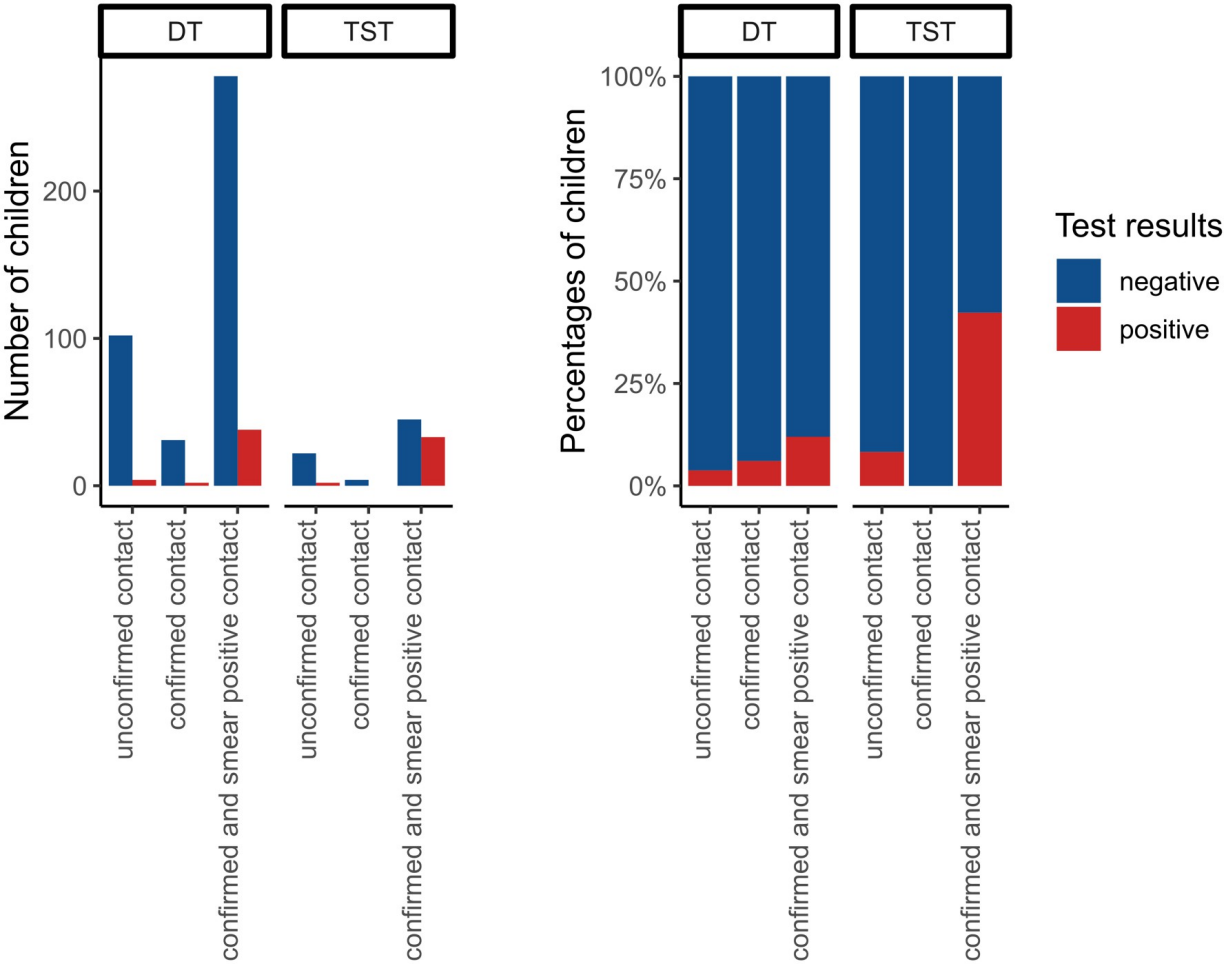
DT and TST positivity in children who had contact to an TB index case, stratified by the smear results of the contact. Children exposed to a smear positive index case were more likely to have a positive DT and TST (in red) compared to children exposed to smear negative or unconfirmed contacts.

Among children referred after contact to an index TB case (n = 457), DT positivity was associated with the contagiousness of the TB index case. In those exposed to a smear positive index case 38/316 (12.1%) were DT positive compared to 2/33 (6.1%) exposed to a smear negative and 4/106 (3.8%) exposed to an unconfirmed TB index case (p = 0.029) (Fig 4).

### DT and TST agreement

A total of 558 children had results for both DT and TST with a median interval between tests of 22 (IQR 13 to 44) days. Test agreement in this group was as follows: DT+/ TST+ in 47 children, DT+/TST- in 3 children, DT-/TST+ in 324 children, TST-/DT- in 184 children (overall agreement 41.4%, 95% CI 37.3 to 45.6, Kappa 0.08, 95% CI 0.013 to 0.14). This was similar for the age group 0–7 years (overall agreement 42.3%, 95% CI 37.6 to 47.1, Kappa 0.06, 95% CI -0.01 to 0.13) and 8–14 years (overall agreement 35.5%, 95% CI 26.5 to 45.3, Kappa 0.07, 95% CI -0.05 to 0.20), and not assessed in the ≥15 years old children with only 9 pairs available.

Test agreement was higher when the time interval between tests was limited to 1, 7, 14 and 21 days with an overall agreement of 63.7% (95% CI 50.1 to 76.0, Kappa of 0.25, 95% CI 0.005 to 0.51, 58 pairs), 54% (95% CI 42.9 to 65.4%, Kappa 0.14, 95% CI -0.06 to 0.35, 81 pairs), 49% (95% CI 41.8 to 57.1, Kappa 0.12, 95% CI -0.005 to 0.25, 176 pairs) and 47% (95% CI 41.6 to 53.9, Kappa 0.11 (95% CI 0.01 to 0.21, 266 pairs), respectively.

## Discussion

This retrospective, observational study details use of DT and TST in a large paediatric population in routine clinical settings in the RF, with a wide range of referral reasons. In our study, few children were diagnosed with TB disease and more were diagnosed with immune correlate of TB infection through routine annual screening. The relatively low number of children with TB disease is possibly related to the timely diagnosis and treatment of TB infection identified by DT and/or TST screening in RF, as a decline in the incidence of TB disease was observed with the establishment of screening in the last decades [9].

Our study shows a high number of children with positive TST, but negative DT results and agreement of the two tests was poor. Children with positive TST are usually referred to the dispensary, at which DT is used as second test to confirm or rule out TB infection. The high rate of children with positive TST and negative DT is likely the result of a higher specificity of the DT compared to TST, even though the precise specificity of DT remains unclear in the absence of a gold standard diagnostic test for TB infection. Importantly, TST has a limited specificity in the setting with almost universal BCG coverage and annual skin testing potentially resulting in a boosting of the test result [23, 24]. We observed an association of positive DT and older age, which likely represents the increasing TB infection prevalence with age. In addition, children referred after TB contact were more likely to have a positive DT compared to children referred for other reasons. This is expected as they have a higher pre-test probability of having a positive test and at the same time suggests good DT sensitivity for TB infection.

These findings are in line with other studies investigating novel skin tests for TB [16]. However, the data comparing the DT with either TST or IGRAs are limited since the DT is only licensed in the RF and some Eastern European countries and IGRAs are not routinely used in the RF. The most recently published systematic review on novel skin tests for TB included 29 studies with DT data [16]. Only four of the studies had a head-to-head comparison of DT against TST or IGRA. Two studies evaluated test agreement and showed a pooled agreement of DT with IGRA of 87.2% which was higher than agreement of DT with TST of 55.5%. Important to note, is that our result of an overall agreement of 41.4% might not be comparable to other studies as a selection bias in our study could have reduced agreement; children with negative TST and/or DT screening are commonly not referred to the TB dispensary and thus the group of children with concordant negative test results are likely to be underestimated in our study. The recently published systematic review also evaluated two studies for DT sensitivity in HIV-uninfected adults with confirmed TB disease and showed a 91% pooled sensitivity which was comparable to the sensitivity of IGRAs and TST. In our study we are unable to calculate sensitivity of the DT since the number of confirmed TB cases was low. The clear advantage of DT or other novel skin tests for diagnosing TB is that neither phlebotomy nor a laboratory is required and if used at large scale these tests are likely to incur lower costs [25] and be more accessible for decentralised care, although like the TST they do require a second clinic visit. This is particularly relevant for public screening approaches especially in high TB-endemic countries.

Our study showed increased prevalence of positive DT results in children exposed to a contact with smear-positive TB, thus a more contagious index case. The same method served also for the validation of IGRA as diagnostic test for TB infection in recent years [26]. Further evaluation of DT through long term follow-up and assessment of progression to TB disease in untreated individuals (with negative and positive DT results) could help to better characterise DT performance [27].

To move TB elimination forward [1, 2], screening for TB infection is and will be even more important in the future and therefore optimal and cost-effective tests as well as screening approaches need to be established. The general risk of progression from TB infection to TB disease within 5 years in individuals without treatment is estimated at 5% [19]. For children risk of progression is much higher: in untreated children <15 years with TB infection the 2-year risk of progression is estimated to be 15% (95% CI, 8–27) and in untreated children <5 years 26% (95% CI, 9–60) [19, 28]. Therefore, children should be a priority population for TB screening.

Our study is of retrospective, observational design which has some important limitations. A significant proportion of children was excluded from our analysis due to unavailability of skin test results. Many of these children were referred for imaging, which is a standard screening approach for TB disease in adolescents in the RF, therefore limiting our analysis in this age group. We were not able to compare the DT with IGRA results, since IGRAs were not routinely done in this setting. We were also unable to estimate the effect of BCG on the DT or TST test results, since very few children were not vaccinated. We were also unable to estimate the effect of repeated DT and TST testing (within the annual screening) on test performance. Finally, results from our study need to be interpreted in the context of the current Russian TB guidelines and screening and treatment practices. Specifically, the interpretation of repeated TST results is specific to the Russian setting as well as the age adapted use of DT and the initiation of treatment for TB infection.

The strength of this study is the high number of children included from a real-world setting where the novel test DT is implemented in routine care. DT is a promising test that could also be suitable for other settings. Our study highlights the challenge in diagnosis of TB infection and the need for well-designed prospective observational studies and randomised trials comparing TB outcomes in children tested with DT and/or TST with and without preventive antimycobacterial treatment.

## Conclusion

In a highly BCG-immunised population children referred for TB screening commonly had a positive TST but were less often positive for DT. This suggests an improved specificity of DT when compared to TST. An association was observed of positive DT and contagiousness of the index case in children who had TB contact, that suggests an improved sensitivity of DT. Further comparisons with IGRAs and prospective studies are warranted.

## Supporting information

S1 Fig Numbers of planned and performed screening tests for children in the Archangelsk region. Numbers of planned and performed screening tests of children ≤14 years of age in the Archangelsk region (RF) by calendar year and type of test were obtained from the regional Ministry of Health. Only aggregated data was available and older adolescents were categorised together with adults. In the Archangelsk region a total of 49771 and 46640 TST, and 31309 and 46089 DT screening tests were done in children aged ≤14 years in 2018 and 2019 respectively. This accounts for 39.1% and 57.2% of planned TST screening, and 41.0% and 42.8% of planned DT screening in the region, respectively. Of the screened children further examination was indicated for 1392 and 1362 children, in 2018 and 2019 respectively, of whom 433 (31.1%) and 507 (37.2%) children aged ≤14 years presented at the TB dispensary.
(TIF)

S2 Fig Reason for referral of children for age groups according to different screening approaches. Children were classified into age groups according to the groups of different screening approaches in the Russian Federation. Colours indicate the reason for referral to the TB dispensary.
(TIF)

S3 Fig DT and TST result in mm for different age groups. Violin and dot-plot of DT and TST in mm for different age groups.
(TIF)

S4 Fig Positive first test result in age groups. Age groups and percentages of positive first test results for DT and TST. On figure *S4A* absolute numbers of children are displayed and on figure *S4B* the same data is displayed as percentages.
(TIF)

S5 Fig Positive first test result stratified by reason for referral. Results of the first DT and first TST stratified by reason for referral to the dispensary.
(TIF)

S1 Table Characteristics of excluded children. Table of characteristics of children who were excluded from the analysis.
(DOCX)

S2 Table Reason for referral and final diagnosis. (DOCX)

S3 Table Univariable and multivariable logistic regression in 2625 children, modelling positivity of the first Diaskintest (DT). Age was associated with positive DT after adjustment for sex.
(DOCX)

## Abbreviations

BCG: Bacillus Calmette-Guérin
CI: Confidence interval
DT: Diaskintest
HIV: Human immunodeficiency virus
IGRA: Interferon-γ release assay
IQR: Inter quartile range
RF: Russian Federation
TB: Tuberculosis
TST: Tuberculin skin test
WHO: World Health Organization
