# Supplementary figures and images for: Diagnosis of tuberculosis infection in children with a novel skin test and the traditional tuberculin skin test: An observational study
Source: PLoS One. 2024 Aug 27;19(8):e0293272. doi: 10.1371/journal.pone.0293272 (PMC11349085; doi:10.1371/journal.pone.0293272)

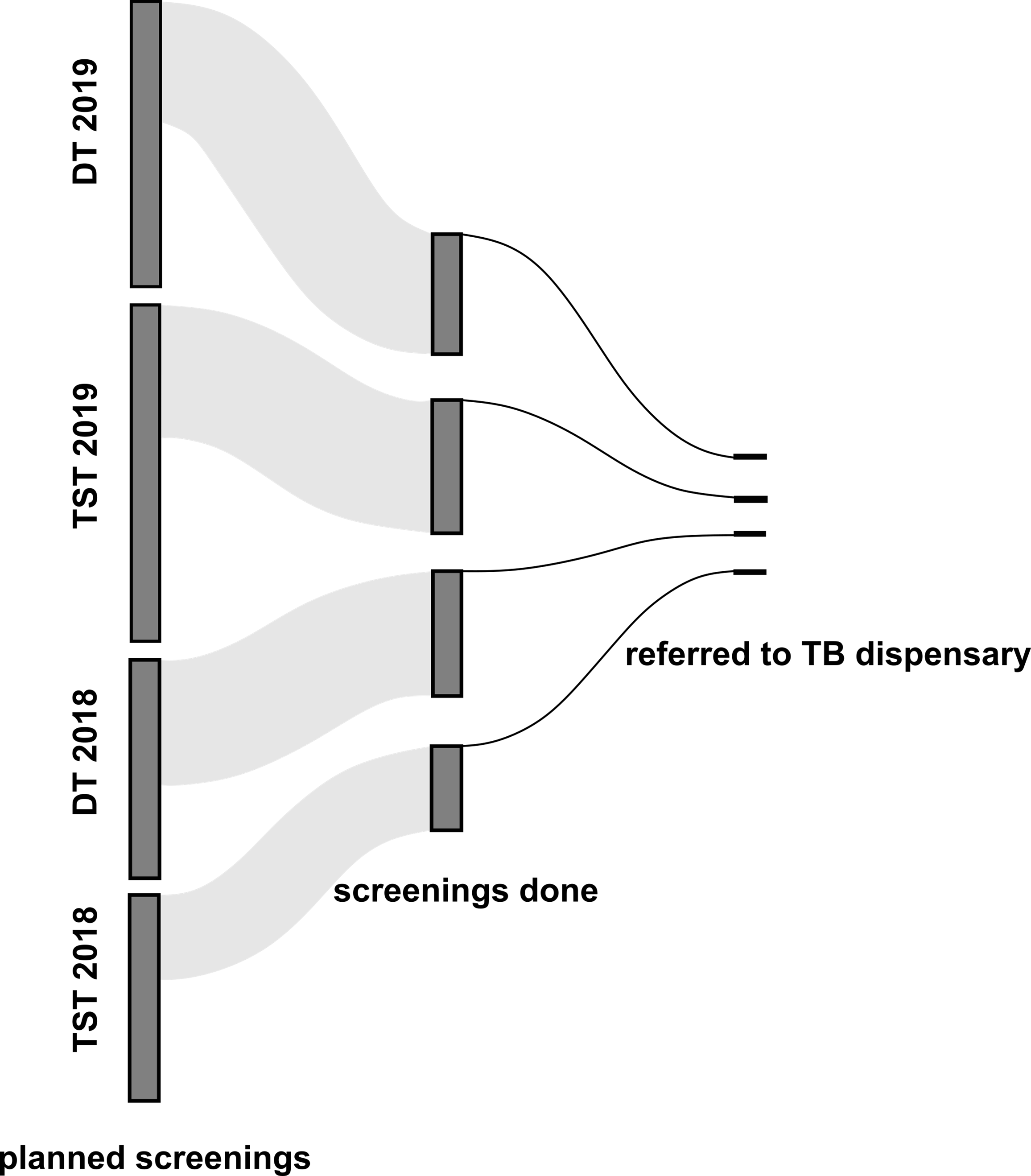

Supplement: S1 Fig — Numbers of planned and performed screening tests of children ≤14 years of age in the Archangelsk region (RF) by calendar year and type of test were obtained from the regional Ministry of Health. Only aggregated data was available and older adolescents were categorised together with adults. In the Archangelsk region a total of 49771 and 46640 TST, and 31309 and 46089 DT screening tests were done in children aged ≤14 years in 2018 and 2019 respectively. This accounts for 39.1% and 57.2% of planned TST screening, and 41.0% and 42.8% of planned DT screening in the region, respectively. Of the screened children further examination was indicated for 1392 and 1362 children, in 2018 and 2019 respectively, of whom 433 (31.1%) and 507 (37.2%) children aged ≤14 years presented at the TB dispensary. (TIF) [file pone.0293272.s001.tif]

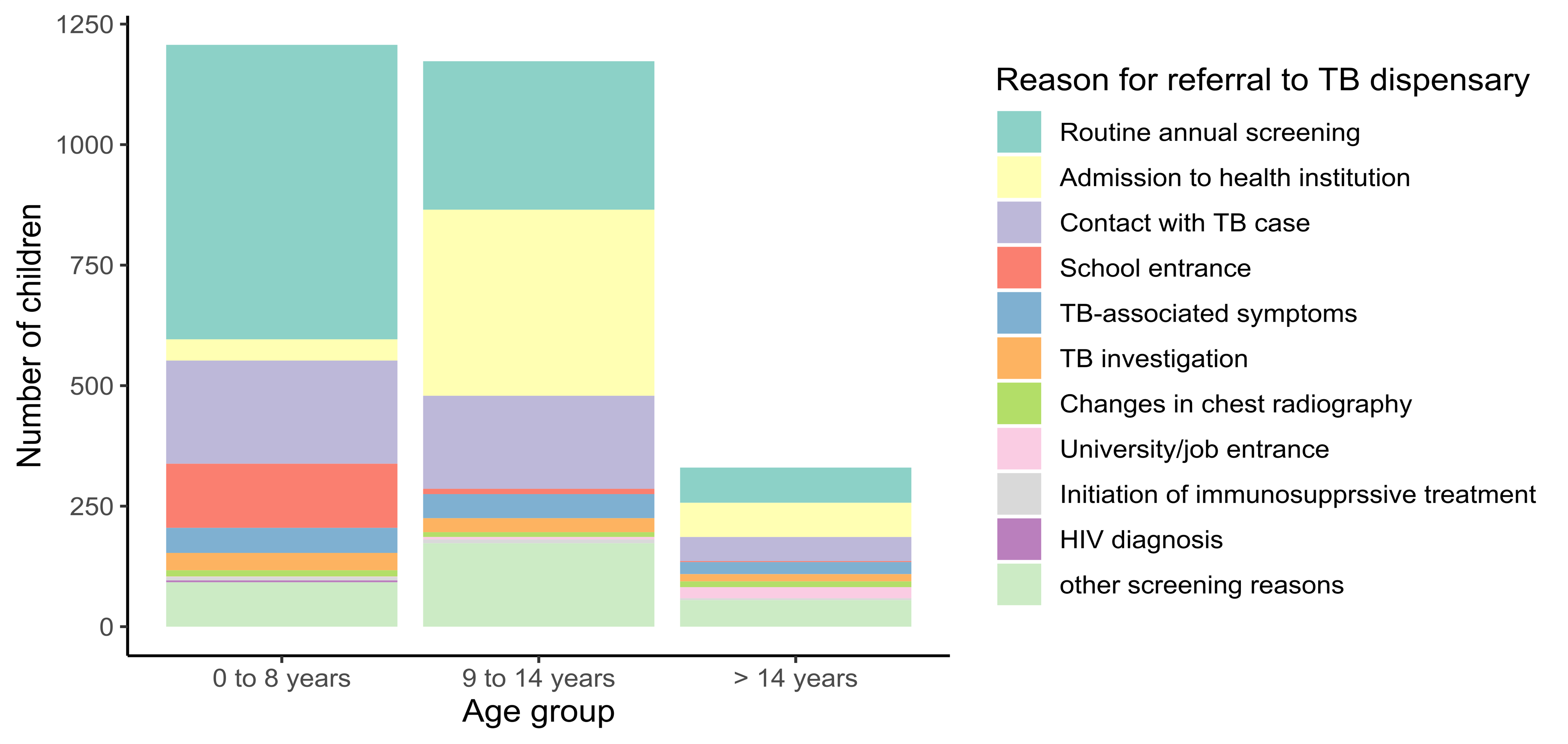

Supplement: S2 Fig — Children were classified into age groups according to the groups of different screening approaches in the Russian Federation. Colours indicate the reason for referral to the TB dispensary. (TIF) [file pone.0293272.s002.tif]

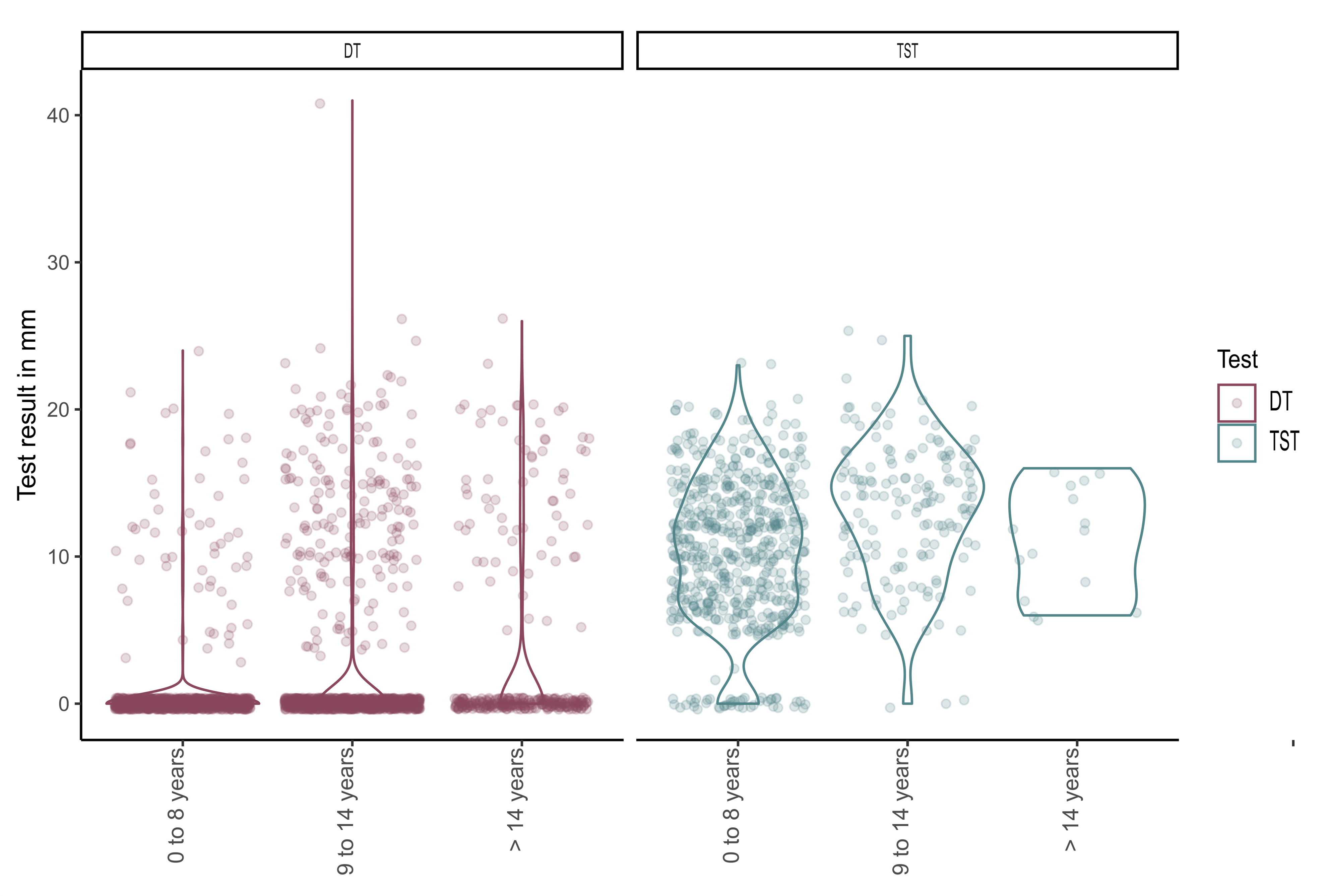

Supplement: S3 Fig — Violin and dot-plot of DT and TST in mm for different age groups. (TIF) [file pone.0293272.s003.tif]

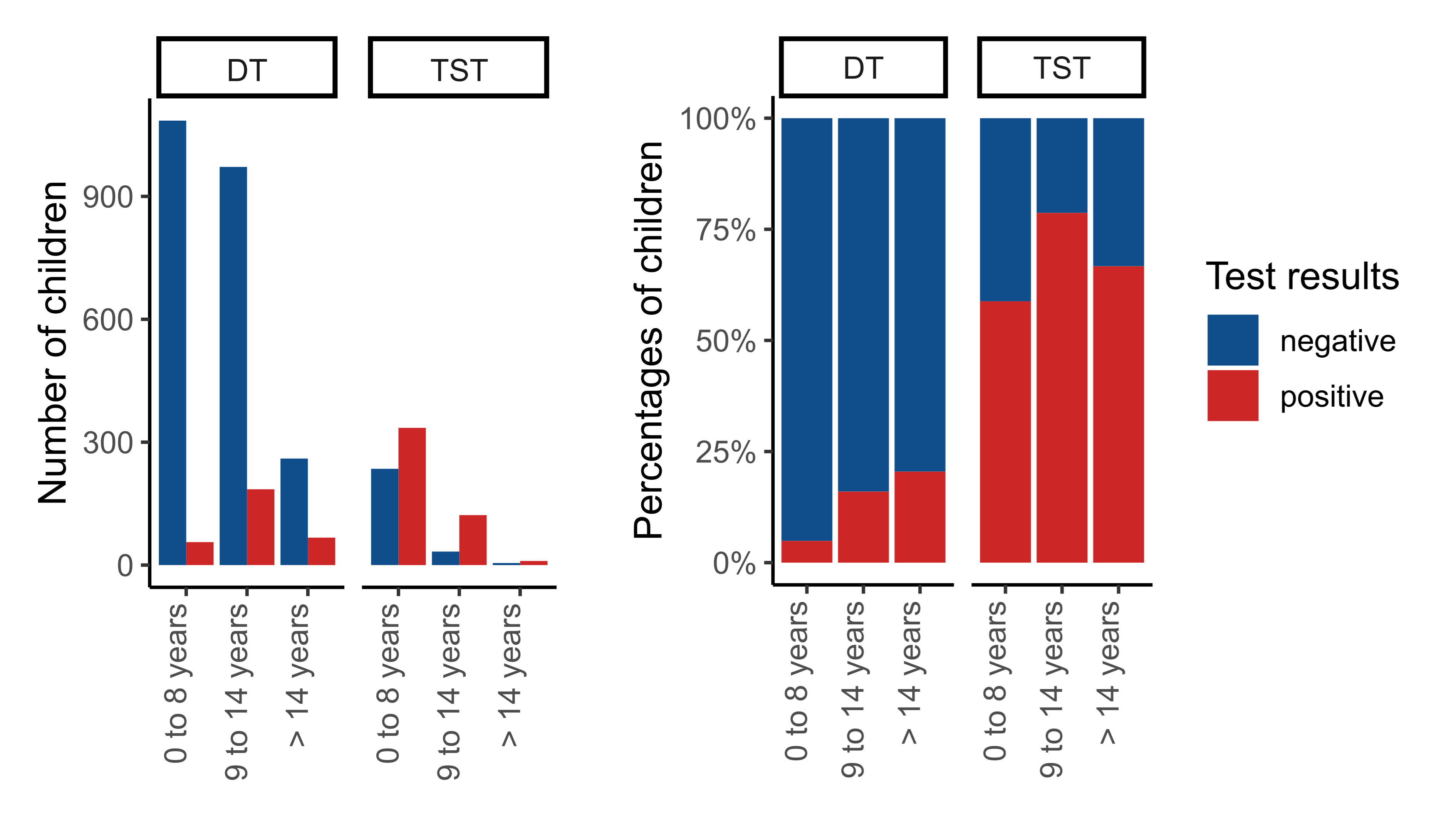

Supplement: S4 Fig — Age groups and percentages of positive first test results for DT and TST. On figure S4A absolute numbers of children are displayed and on figure S4B the same data is displayed as percentages. (TIF) [file pone.0293272.s004.tif]

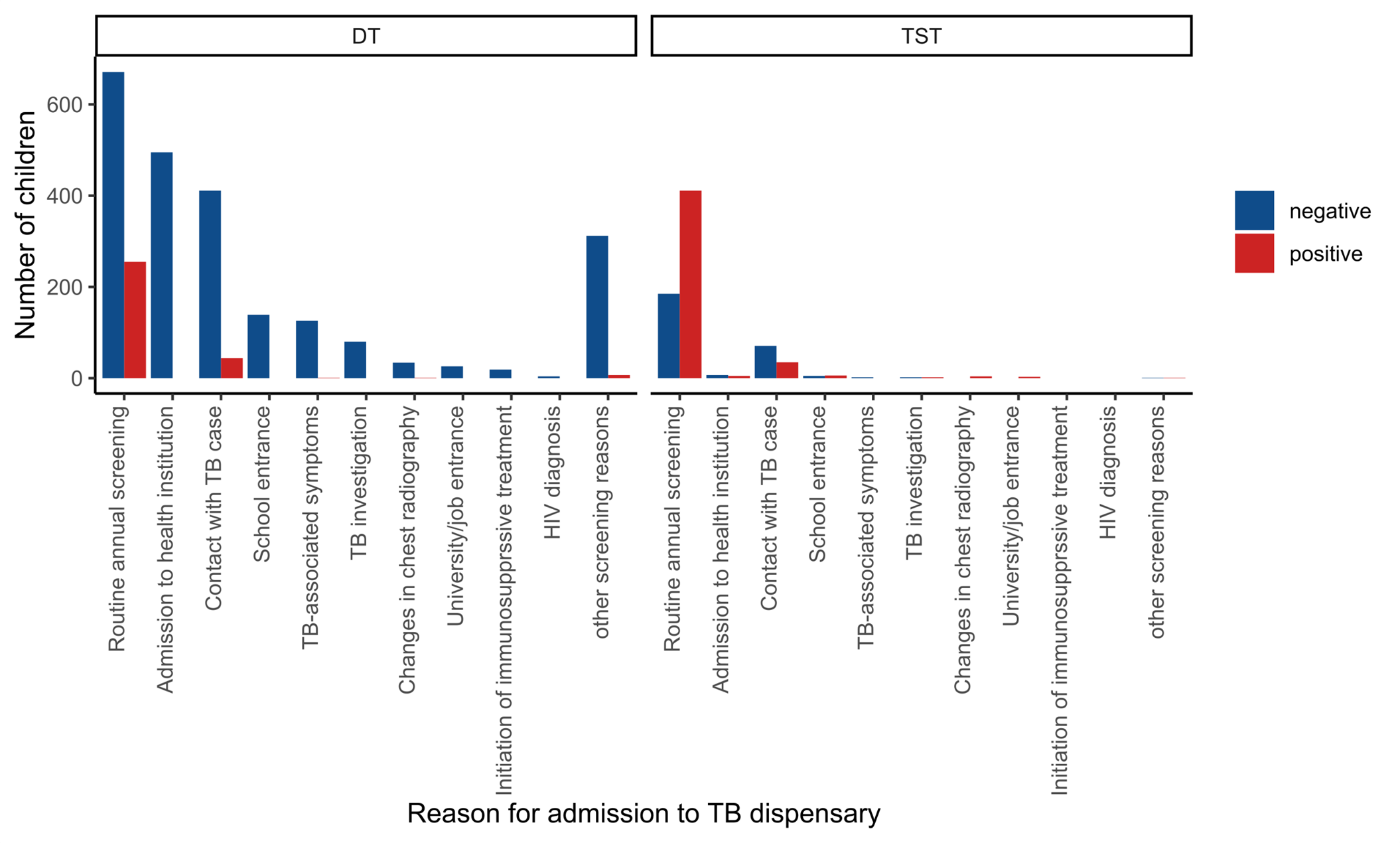

Supplement: S5 Fig — Results of the first DT and first TST stratified by reason for referral to the dispensary. (TIF) [file pone.0293272.s005.tif]
