# Supplementary material for: Diagnosis of tuberculosis infection in children with a novel skin test and the traditional tuberculin skin test: An observational study
Source: PLoS One. 2024 Aug 27;19(8):e0293272. doi: 10.1371/journal.pone.0293272 (PMC11349085; doi:10.1371/journal.pone.0293272)
Supplement: S1 Table — Table of characteristics of children who were excluded from the analysis. (DOCX) [file pone.0293272.s006.docx]

**S1 Table:** Table of characteristics of children who were excluded from the analysis.

|  | Referred for BCG complication  (N=50) | No immunodiagnostic test  (N=1061) | No immunodiagnostic test in study time (N=105) | No valid immunodiagnostic test (N=31) | Total  (N=1247) |
| --- | --- | --- | --- | --- | --- |
| **Age [years], Median (IQR**) | 0.8 (0.4 – 2.5) | 10.2 (3.4 – 15.9) | 12.2 (6 – 16.3) | 10.9 (8.1 – 12.9) | 9.9 (3.3 – 15.9) |
| **Age groups** |  |  |  |  |  |
| 0 to 8 years | 48 (96.0%) | 450 (42.4%) | 38 (36.2%) | 8 (25.8%) | 544 (43.6%) |
| 9 to 14 years | 2 (4.0%) | 242 (22.8%) | 26 (24.8%) | 19 (61.3%) | 289 (23.2%) |
| ≥ 15 years | 0 (0.0%) | 369 (34.8%) | 41 (39.0%) | 4 (12.9%) | 414 (33.2%) |
| **Year** |  |  |  |  |  |
| 2018 | 25 (50.0%) | 424 (40.0%) | 22 (21.0%) | 9 (29.0%) | 480 (38.5%) |
| 2019 | 25 (50.0%) | 637 (60.0%) | 83 (79.0%) | 22 (71.0%) | 767 (61.5%) |
| **Sex**: **male** | 34 (68.0%) | 630 (59.4%) | 68 (64.8%) | 14 (45.2%) | 746 (59.8%) |
| **BCG vaccination** |  |  |  |  |  |
| 0 | 1 (2.0%) | 128 (12.6%) | 2 (2.0%) | 1 (3.2%) | 132 (11.0%) |
| 1 | 45 (90.0%) | 873 (85.9%) | 96 (97.0%) | 30 (96.8%) | 1044 (87.3%) |
| 2 | 4 (8.0%) | 12 (1.2%) | 1 (1.0%) | 0 (0.0%) | 17 (1.4%) |
| At least one, but number unknown | 0 (0.0%) | 3 (0.3%) | 0 (0.0%) | 0 (0.0%) | 3 (0.3%) |
| Unknown | 0 | 45 | 6 | 0 | 51 |
| **Main reason for referral** |  |  |  |  |  |
| Admission to health institution | 0 (0.0%) | 320 (30.2%) | 18 (17.1%) | 3 (9.7%) | 341 (27.3%) |
| School entrance | 0 (0.0%) | 311 (29.3%) | 8 (7.6%) | 0 (0.0%) | 319 (25.6%) |
| University/job entrance | 0 (0.0%) | 275 (25.9%) | 30 (28.6%) | 0 (0.0%) | 305 (24.5%) |
| Abnormal annual screening | 0 (0.0%) | 6 (0.6%) | 28 (26.7%) | 28 (90.3%) | 62 (5.0%) |
| BCG related reason | 50 (100.0%) | 0 (0.0%) | 0 (0.0%) | 0 (0.0%) | 50 (100%) |
| TB-associated symptoms | 0 (0.0%) | 32 (3.0%) | 6 (5.7%) | 0 (0.0%) | 38 (3.0%) |
| TB investigation | 0 (0.0%) | 13 (1.2%) | 1 (1.0%) | 0 (0.0%) | 14 (1.1%) |
| Contact with TB case | 0 (0.0%) | 7 (0.7%) | 2 (1.9%) | 0 (0.0%) | 9 (0.7%) |
| Changes in chest radiography | 0 (0.0%) | 2 (0.2%) | 1 (1.0%) | 0 (0.0%) | 3 (0.2%) |
| Other screening reasons | 0 (0.0%) | 95 (9.0%) | 11 (10.5%) | 0 (0.0%) | 106 (8.5%) |
| **Status at end of study time** |  |  |  |  |  |
| Followed at dispensary | 10 (20.0%) | 4 (0.4%) | 2 (1.9%) | 5 (16.1%) | 21 (1.7%) |
| Discharged | 39 (78.0%) | 961 (90.6%) | 99 (94.3%) | 26 (83.9%) | 1125 (90.2%) |
| Lost to follow-up | 1 (2.0%) | 96 (9.0%) | 4 (3.8%) | 0 (0.0%) | 101 (8.1%) |
| **Chest radiography reported** | 11 (22.0%) | 841 (79.3%) | 68 (64.8%) | 27 (87.1%) | 947 (75.9%) |
| **Computed tomography reported** | 0 (0.0%) | 16 (1.5%) | 6 (5.7%) | 5 (16.1%) | 27 (2.2%) |
