# Supplementary material for: Diagnosis of tuberculosis infection in children with a novel skin test and the traditional tuberculin skin test: An observational study
Source: PLoS One. 2024 Aug 27;19(8):e0293272. doi: 10.1371/journal.pone.0293272 (PMC11349085; doi:10.1371/journal.pone.0293272)
Supplement: S2 Table — (DOCX) [file pone.0293272.s007.docx]

**S2 Table**: Reason for referral and final diagnosis

|  | Abnormal annual screening (N=992) | Admission to health institution (N=501) | Contact with TB case (N=457) | School entrance (N=146) | | TB-associated symptoms (N=127) | TB investigation (N=80) | Changes in chest radiography (N=35) | University/job entrance (N=29) | Initiation of immunosuppressive treatment (N=19) | HIV diagnosis (N=4) | other screening reasons (N=320) | Total (N=2710) |
| --- | --- | --- | --- | --- | --- | --- | --- | --- | --- | --- | --- | --- | --- |
| TB uninfected, untreated | 371 (37.4%) | 495 (98.8%) | 325 (71.1%) | | 140 (95.9%) | 125 (98.4%) | 78 (97.5%) | 29 (82.9%) | 26 (89.7%) | 19 (100.0%) | 4 (100.0%) | 311 (97.2%) | 1923 (71.0%) |
| TB uninfected, prophylactically treated | 2 (0.2%) | 0 (0.0%) | 51 (11.2%) | | 0 (0.0%) | 0 (0.0%) | 0 (0.0%) | 0 (0.0%) | 0 (0.0%) | 0 (0.0%) | 0 (0.0%) | 0 (0.0%) | 53 (2.0%) |
| Immune correlate of TB infection, untreated | 467 (47.1%) | 6 (1.2%) | 26 (5.7%) | | 6 (4.1%) | 1 (0.8%) | 2 (2.5%) | 5 (14.3%) | 3 (10.3%) | 0 (0.0%) | 0 (0.0%) | 8 (2.5%) | 524 (19.3%) |
| Immune correlate of TB infection, treated | 151 (15.2%) | 0 (0.0%) | 48 (10.5%) | | 0 (0.0%) | 0 (0.0%) | 0 (0.0%) | 0 (0.0%) | 0 (0.0%) | 0 (0.0%) | 0 (0.0%) | 1 (0.3%) | 200 (7.4%) |
| TB disease | 1 (0.1%) | 0 (0.0%) | 7 (1.5%) | | 0 (0.0%) | 1 (0.8%) | 0 (0.0%) | 1 (2.9%) | 0 (0.0%) | 0 (0.0%) | 0 (0.0%) | 0 (0.0%) | 10 (0.4%) |
