# Supplementary material for: Diagnosis of tuberculosis infection in children with a novel skin test and the traditional tuberculin skin test: An observational study
Source: PLoS One. 2024 Aug 27;19(8):e0293272. doi: 10.1371/journal.pone.0293272 (PMC11349085; doi:10.1371/journal.pone.0293272)
Supplement: S3 Table — Age was associated with positive DT after adjustment for sex. (DOCX) [file pone.0293272.s008.docx]

**S3 Table:** Univariable and multivariable logistic regression in 2625 children, modelling positivity of the first Diaskintest (DT). Age was associated with positive DT after adjustment for sex.

| Variable | univariable | | | multivariable | | |
| --- | --- | --- | --- | --- | --- | --- |
|  | **OR** | **95% CI** | **p** | **OR** | **95% CI** | **p** |
| Age (per year increase) | 1.16 | 1.13 – 1.19 | <0.001 | 1.16 | 1.13 - 1.19 | <0.001 |
| Sex (female) | 1.21 | 0.95 - 1.53 | 0.12 | 1.23 | 0.97 – 1.57 | 0.089 |
